# Not by Selection Alone: Expanding the Scope of Gene‐Culture Coevolution

**DOI:** 10.1002/evan.70007

**Published:** 2025-07-19

**Authors:** Sven M. Kasser, Kevin N. Lala, Laura Fortunato, Marcus W. Feldman

**Affiliations:** ^1^ School of Biology University of St Andrews St Andrews UK; ^2^ Institute of Human Sciences University of Oxford Oxford UK; ^3^ Santa Fe Institute Santa Fe USA; ^4^ Department of Biology Stanford University Stanford California USA

**Keywords:** cultural evolution, dual‐inheritance theory, gene‐culture coevolution, human evolution, population genetics, social learning

## Abstract

Gene‐culture coevolution (GCC)—an ambitious synthesis of biological and social sciences is often used to explain the evolution of key human traits. Despite the framework's broad conceptual appeal however, empirical evidence is often perceived as limited to a few key examples like lactase persistence. We argue this apparent gap between theoretical appeal and empirical evidence stems from conceptual ambiguities regarding the scope of relevant gene‐culture interactions. Drawing on recent work in animal gene‐culture coevolution and human genomics, we propose a “broad” approach that formally incorporates drift and migration alongside natural selection. This builds upon and subsumes the existing “narrow” framework focused on selection. Through case studies of skin pigmentation evolution and gift‐exchange network influences on genetic variation in Melanesia, we demonstrate how cultural factors shape both adaptive and neutral genetic variation and population structure. This integrative perspective accommodates diverse empirical findings while opening new avenues for research in human evolution.

## 1 Introduction

A new perspective on human evolution has emerged over the past 50 years that attempts to integrate insights from the “natural” and “social” sciences into a comprehensive formal framework that can explain the distinctive evolutionary trajectory of the human species. Variously labeled as “gene‐culture coevolution” ([1], used exclusively hereafter), “culture‐gene coevolution” [2] or “dual inheritance theory” [3], this approach posits that genes and culture represent two separate, yet deeply intertwined, inheritance systems that often result in (adaptive, but occasionally maladaptive) phenotypic change over time [4, 5, 6, 7, 8, 9, 10, 11].

That “species‐defining” human phenotypes—for example our capacity for language, technology, cooperation, and complex problem‐solving—are not solely determined by genetic variation, but subject to a rich causal mosaic of interacting factors, including the cultural environment, is widely accepted [12, 13, 14]. The study of such interactions is not exclusive to gene‐culture coevolutionary theory; it is also important, for example, in the quantification of gene‐environment interactions and the analysis of correlations between relatives [15, 16]. However, the evolutionary significance of this interplay can be elucidated through a theoretically rigorous and empirically grounded science of gene‐culture coevolution.

The central concepts of gene‐culture coevolution have changed relatively little since its inception through the pioneering work of Cavalli‐Sforza and Feldman [4, 7, 11], and later Boyd and Richerson [3]. At its core, it shares with broader cultural evolutionary theory a commitment to the idea that the population‐level transmission dynamics of cultural information mimic those of biological evolution sufficiently to allow for an evolutionary framework of cultural change [17]. But beyond that, it focuses on the possible *interactions* between the two systems of inheritance, that is, co‐dependence of genotype and cultural phenotype. For example, the term “gene‐culture coevolution” is often applied to instances where, by modifying ecological conditions, cultural traits shape the form of natural selection acting on the population's genome (a process known as cultural niche construction, see for example, [18, 19, 20]).

Our understanding of gene‐culture coevolution is typically closely aligned with the traditional use of the term “coevolution” in evolutionary ecology. In a now‐classic paper, titularly asking “When is coevolution?” Janzen [21, p. 611] defines coevolution as “an evolutionary change in a trait of the individuals in one population in response to a trait of the individuals of a second population, followed by an evolutionary response by the second population to the change in the first.” Even though Janzen himself did not specify *adaptive* evolutionary change, he does credit an earlier paper by Ehrlich and Raven [22, p. 606] with establishing the field of coevolution, who in turn specifically highlight “reciprocal selective responses.” Indeed, natural selection is often invoked as the primary mechanism generating coevolutionary dynamics [23], and the terms of “coevolution” and “coadaptation” are usually used interchangeably within evolutionary ecology [24]. However, Janzen's focus on trait‐level coevolution, specifically, has been subject to revision [23, 25]. In a recent review, Dixit [26, p. 212] characterizes coevolution somewhat more broadly as “reciprocal adaptive evolution at any level of biological organisation.” Three key elements of this definition are worth drawing out here: coevolution is (a) *reciprocal*, that is causally bidirectional (echoing [21]), (b) *adaptive*, i.e., primarily effected through mechanisms of selection and adaptation (and thus, effectively collapsing coevolution and coadaptation), and (c) *multilevel*, that is, not limited to inter‐specific evolutionary interactions. This useful contemporary definition moves away from more traditional ones (e.g., [21, 24]) by explicitly allowing for coevolutionary dynamics beyond genes as the primary substrate of coevolution and inter‐specific interactions as its primary arena. This is to say that even evolutionary ecology is moving away from privileging “classic” dynamics between predators and prey, or hosts and pathogens, as the sole focus of coevolutionary thinking. However, like those earlier definitions, it invokes a deep causal interdependence between the coevolving elements—or, more precisely, *dynamic reciprocal evolutionary change*, usually in the form of specific *coadaptation* marked by reciprocal influences on the relative fitness of interacting traits and specific phenotypes [23].

Accordingly, a key aspect of gene‐culture *coevolution* theory is that biological and cultural evolutionary processes interact through mutual causal feedback. For instance, biologically rooted predisposition toward certain relevant phenotypes (e.g., evolved perceptual, motivational or cognitive biases) may shape what and how cultural information is acquired, stored and transmitted. At the same time, cultural practices can modify the ecological conditions that drive natural selection on the human genome, including genotypes underlying the traits that enable and amplify enculturation in the first place [27, 28]. To account for this interplay between often disparate domains of inquiry, the science of gene‐culture coevolution must draw upon a wide range of relevant disciplines, from genetics to cognitive science and cross‐cultural anthropology.

Such an ambitious scientific endeavor requires conceptual clarity. In the present paper, we set out to both clarify and expand what researchers traditionally mean by “gene‐culture coevolution.” We first query the central scientific motivations behind applying gene‐culture coevolutionary frameworks and define *narrow gene‐culture coevolution*, namely the more traditional and commonly applied approach, before subsequently introducing an expansion of that framework, which we call *broad gene‐culture coevolution*. This expanded framework incorporates nonselective mechanisms, namely drift and migration, into gene‐culture coevolutionary theory, which produces a more comprehensive explanatory framework for analyzing recent human evolution. We illustrate the utility of this wider viewpoint with two examples of the way culture interacts with these processes: the buffering role of cultural traits in the adaptive evolution of skin pigmentation, and the way that migration by way of a traditional trading network, the Kula ring, may have shaped genetic patterns in Oceania. We conclude by considering the limitations and boundaries of this expanded approach and suggesting a way forward for the wider field.

## 2 Modes of Gene‐Culture Coevolution

### 2.1 Narrow Gene‐Culture Coevolution

Many common definitions of gene‐culture coevolution mirror, explicitly or implicitly, the coevolution through‐coadaptation conception that dominates evolutionary ecology. Their originators, among them many of the field's key contributors, appear primarily concerned with gene‐culture interactions that lead to “new selective pressures” [10, p. 8985], to “selection […] generated or modified by [culture]” [1, p. 453], or that become the “basis for genetic selection” [29, p. 879]. In other words, the focus clearly appears to be on *selective* processes, namely on cases where cultural elements modify the natural selection of genetic variation (the “culture‐to‐genes” direction of gene‐culture coevolution), but where the requirement of reciprocal co‐adaptation—that is, cases where frequencies of associated cultural and genetic variants change together—appears to have been relaxed (see also [30]).

**Figure 1:**
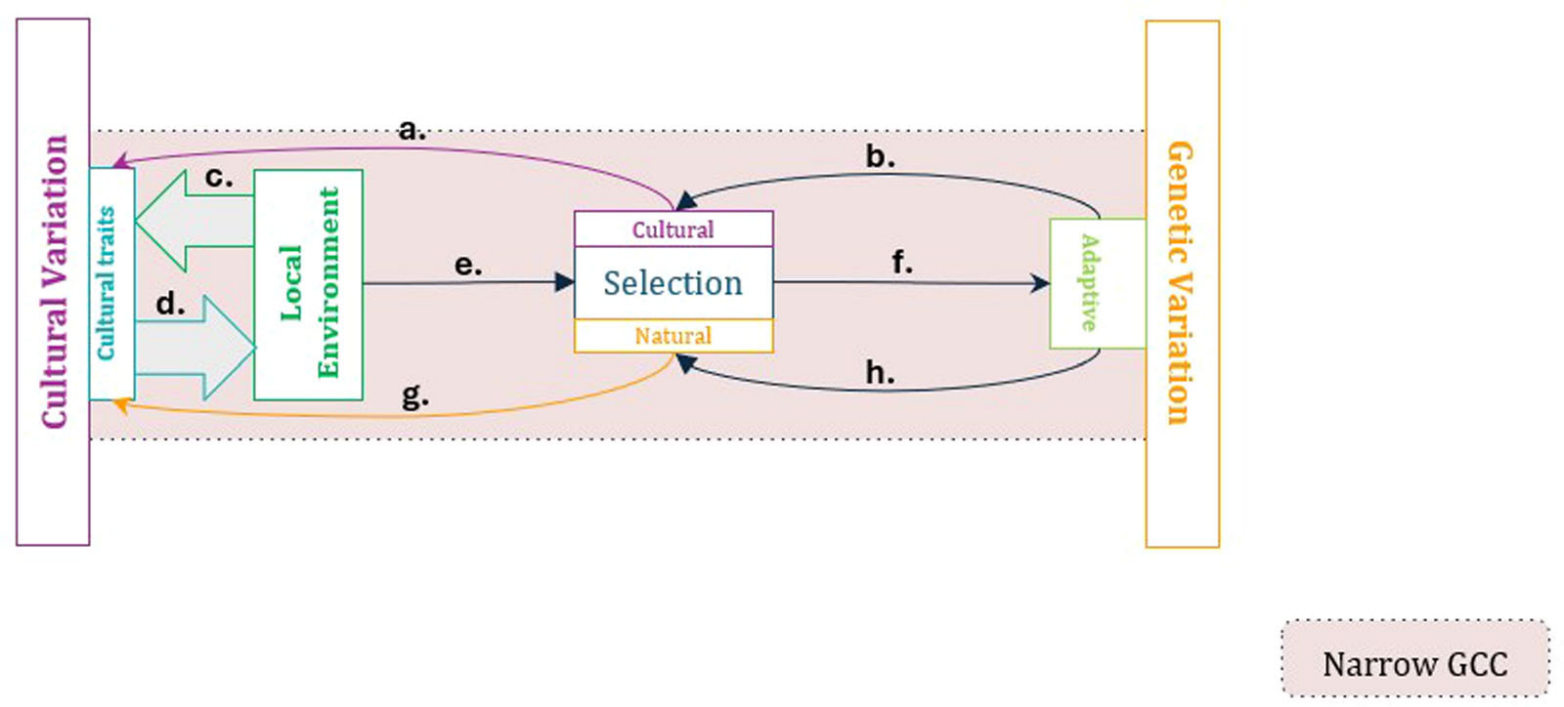
Causal diagram for narrow gene‐culture coevolution. The arrows here represent the following causal interactions: (a) cultural selection on cultural variation (i.e., the differential transmission and copying of cultural variants, e.g., due to conformist bias), (b) genetically evolved (cognitive) biases in social learning, (c) population‐level cultural adaptation (through individual‐level learning), (d) cultural niche construction, (e) the eco‐cultural environment as a source of selection, (f) natural selection of (adaptive) genetic variation, (g) natural selection of cultural variation, (h) genetic and developmental biases in the natural selection of cultural variation (e.g., evolved anatomical morphology, lactase persistence genotypes, etc.).

*Narrow* gene‐culture coevolution focuses on the reciprocal action of selective processes on both cultural and genetic variation (see Figure 1). Culturally evolved traits and the local ecology can interact to form the selective environment (including tangible and intangible features, e.g., physical geography and social norms). In principle, this interaction is inherently reciprocal. Some cultural traits are adaptive and shaped by the local ecology [31, 32]. At the same time, culturally evolved traits can profoundly shape both the local ecology itself (e.g., choosing or modifying the landscape) and the impact of ecological variation (e.g., buffering against seasonality), a phenomenon known as “niche construction” [33, 34, 35]. While the dynamics of these interactions are important topics of research in themselves, the gene‐culture coevolutionary framework primarily concerns their outcome, namely, the eco‐cultural “landscape” that sets the stage for genetic selection, which affects adaptive genetic variation. Take the flagship example of gene‐culture coevolution, lactase persistence (i.e., the ability of humans to digest lactose into adulthood; see Box 1). Here, the eco‐cultural dynamics of dairying practices and availability of grazing land lead to presence or absence (or varying extent) of dairy pastoralism, which in turn determines the fitness advantage of lactase persistence‐related alleles, whose spread, in turn, may affect the incidence and utility of dairying.

BOX 1 **Cultural modification of selection.**

The best known example of narrow sense gene‐culture coevolution is the evolution of human adult lactose tolerance and the associated ability to digest dairy products. Most humans (and most mammals) lose the ability to digest lactose, a disaccharide sugar that forms the principal component of milk, as they mature [36]. However, some populations have retained this ability, termed lactase persistence, due to genetic variants in the lactase gene LCT and associated regulatory regions such as MCM6 [37, 38, 39, 40]. These populations also tend to have a strong cultural history of cattle farming and milk consumption, giving rise to the now‐classic gene‐culture coevolutionary hypothesis that dairy farming, as a cultural practice that provides a novel abundant source of milk in the diet, may predate this adaptation and may have produced the selection pressure to favor lactase persistence [41, 42, 43, 44, 45, 46]. This example also showcases the potentially transformative power of gene‐culture coevolution, as selection on LCT is estimated to have been some of the strongest selection in the human genome to date [47]. While this represents a textbook example of gene‐culture coevolution (e.g., [6]), not all details of the lactase persistence story are fully understood, and important aspects (e.g., the strength, timing, and mechanisms of selection) are continually revisited and updated as new ancient DNA is analyzed and archaeological evidence emerges [48, 49]. For example, Evershed et al. [50] have noted that selection of lactase persistence alleles does not seem to correlate with the level of milk exploitation (arguably the cultural trait in question), highlighting the complexity of gene‐culture coevolutionary inference even in the narrow sense. Furthermore, there is the distinct possibility that a two‐trait model of gene‐culture coevolution is insufficient for understanding even this example, as evidence suggests some populations may have adapted culturally by reducing the lactose content of milk through further processing (e.g., fermentation into yogurt [51];) or developmentally through changes to the microbiome [52], rather than genetically via lactase persistence. Nevertheless, the evolution of lactase persistence is remarkable in the wide attention and general acceptance it has received in the scientific community as a cross‐disciplinary phenomenon [39, 50], spanning genomics, medicine, anthropology, and archaeology. This prominence has played a key role in reifying the belief that narrow gene‐culture coevolution is not just a hypothetical possibility, leading geneticists and interdisciplinary teams to propose other candidate cases of gene‐culture coevolution (albeit only rarely in the terms of that theory). For example, among indigenous Inuit populations of Greenland, there is evidence for extensive genetic adaptation to diets with a high content of polyunsaturated fats (or PUFAs), which has been posited to be a direct function of the predominantly fishing‐based subsistence of these Arctic marine hunters [53, 54]. Elsewhere, human dietary adaptations have been proposed for regulating the intake of a number of other macro‐ and micro‐nutrients, including starch Perry et al. [55], iron [56], calcium [57], zinc [58], and selenium [59]—the relative abundance of each of which in human diets is often directly dependent on the subsistence culture [60]. Local adaptation may also have shaped physiological responses to dietary by‐products and toxins. Genes coding for alcohol dehydrogenase (ADH), an enzyme involved in the detoxification and processing of alcohol, exhibit remarkable geographic variation and signatures of recent selection [61, 62]; some variants may result in a protective genetic response to cultural practices such as rice cultivation in East Asia, which gave rise to increased alcohol consumption [63]. Recent studies of ancient DNA suggest that the number of copies of the amylase gene may also have increased as a consequence of human adoption of agriculture [64, 65].

More generally, the genetic variation favored by culturally modified natural selection may feedback to shape the selection acting on cultural variation in at least two ways. On the one hand, both cognitive biases towards certain cultural traits [66, 67] and a psychological bias towards cultural learning [27, 68, 69] may evolve and shape the action of cultural selection, namely the biased transmission and spread of certain cultural variants [9]. On the other hand, genetic contributions may also influence the fitness, and hence natural selection of individuals with certain cultural traits, or certain variants of a particular cultural trait. In the lactase persistence example, the presence or absence of lactase persistence may have modified the fitness advantage of practicing dairy pastoralism [44]. Taken together with the culturally shaped selection on genotypes, these two pathways produce the reciprocal feedback fundamental to the classic gene‐culture coevolutionary approach (see Figure 1).

With its focus on selection, this narrow mode of gene‐culture coevolution is often invoked as a framework to explain the form and function of evolved cultural and biological traits, as well as their perceived distinctiveness. In this context, such a selection‐focused approach can certainly be productive. Gene‐culture coevolutionary approaches to explain such phenotypes as lactase persistence (see Box 1) or even large‐scale cooperation and musicality, employ gene‐culture coevolutionary theory in this fashion [2, 29, 70].

### 2.2 Broad Gene‐Culture Coevolution

Our key contention is that if, conversely, the aim of gene‐culture coevolutionary research is to elucidate the wider evolutionary dynamics of species with a rich cultural capability, humans chiefly among them, and specifically the causal role culture plays in influencing these dynamics, then a *broader* array of gene‐culture interactive processes needs to be considered to fully account for the emergence of and change in genetic and cultural variation. In their initial formulation of the cultural evolutionary theory in which gene‐culture coevolution is rooted, Cavalli‐Sforza and Feldman [11] extensively model the role that neutral evolutionary processes like drift and migration, rather than selection alone, play in shaping not only genetic, but also cultural evolution. However, subsequent works that can be seen as early approaches to gene‐culture coevolutionary theory de‐emphasize drift and migration as core mechanisms [3, 6], and largely adopt the narrow approach outlined above. Only recently has the importance of drift and migration in the explicit context of gene‐culture coevolution again been highlighted, notably in work on nonhuman animals, such as whales, where the influence of culture on both adaptive and neutral (i.e., nonselective) evolutionary processes is emphasized [30].

Why is it important to adopt a similar approach in regard to human gene‐culture coevolution? As we describe below, there are strong empirical reasons to do so. Genetic analyses suggest that human biological evolution since the emergence of our species is profoundly affected, perhaps dominated, by drift, founder effects, and gene flow, rather than (positive) natural selection [71, 72, 73]. This implies that a focus on selection alone is unlikely to reveal the full picture, and that other evolutionary processes, including (cultural impacts on) mutation, drift, and gene flow, should be included. In fact, it seems highly plausible that narrow gene‐culture coevolution may inadvertently exclude some of the most important forms of evolutionary interaction between genes and culture in humans.

Additionally, without retreading the adaptationism debates of the 70 and 80s (but see [74], for a helpful review), it is important, if perhaps obvious, to consider that if gene‐culture coevolutionary approaches only *look* for adaptation, they will only *find* adaptation (or a lack thereof). That is to say, any evidence generated in a “narrow” gene‐culture coevolution framework will frame the evolutionary dynamics of a given trait primarily in light of selection. As other have argued, there are distinct (practical) limits to such an adaptation‐focused approach in explanations of human phenotypes [75]. Furthermore, such critiques are certainly not unique to the context of human gene‐culture coevolution, but embedded in a broader conceptual discourse in the natural sciences. Indeed, neutralist‐selectionist debates [76] and disagreements about the role of “constructive neutral evolution” [77] in the evolution of traits and species pervade the field of evolutionary biology more broadly, and remain largely unresolved [78].

And yet, given such schisms, it is worth noting here that our approach does not reject what we have termed “narrow gene‐culture coevolution,” but rather subsumes it. A broad approach to gene‐culture coevolution does not ignore selection, nor does it eschew adaptation—it merely places these phenomena in a wider evolutionary context. In short, we believe that approaches to gene‐culture coevolution may differ in *scope* (emphasizing selection alone vs emphasizing selection, drift, and migration) because the application of the framework may differ in *purpose* (explaining traits vs. characterizing wider evolutionary dynamics and the causal role of culture in this context).

**Figure 2:**
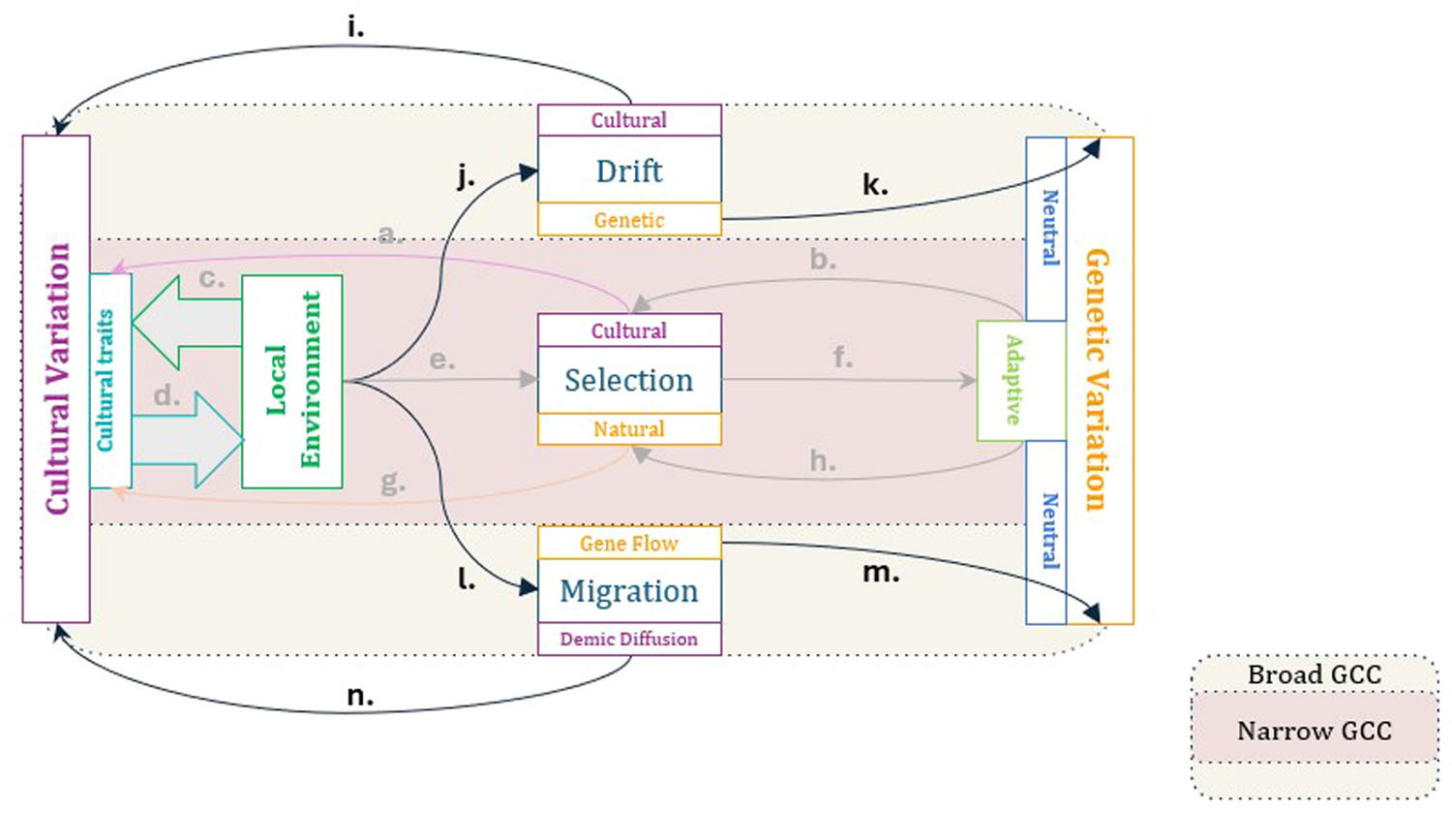
Causal diagram for broad gene‐culture coevolution. In addition to the processes outlined in Figure 1, this framework includes the following: (i) cultural drift, (j) eco‐cultural and demographic background conditions of genetic drift (e.g., environmental carrying capacity and population size), (k) genetic drift, (l) eco‐cultural and demographic background conditions of migration (e.g., geographical barriers and the cultural knowledge needed to overcome them), (m) gene flow, (n) demic diffusion (i.e., movement of cultural traits through movement of people).

As such, broad gene‐culture coevolution accepts all the core gene‐culture interactions of the narrow framework but also incorporates two additional evolutionary mechanisms that contribute to both cultural and genetic variation: drift and migration (see Figure 2). Eco‐cultural dynamics may interact with selection, but can also shape gene and cultural trait frequency changes that arise through drift and migration. There is strong molecular evidence to suggest that such neutral evolutionary forces have played a major role in shaping extant patterns of human genetic variation [11, 71, 72, 73, pp. 109–124, 157–179]. This is not to say that selection, both positive selective sweeps and background selection, is unimportant in explaining genetic variation and adaptation in humans [79, 80, 81, 82, 83]. Some studies even suggest that recent linked positive selection (i.e., selection on genomic regions that are physically adjacent to actual targets of selective sweeps) may affect as much as half of the human genome [81], though subsequent analyses have called this result into question [84, 85], and other estimates are much more conservative, at 10% or less [73]. Similarly, background selection (i.e., purifying selection acting on deleterious variants) may lead to a substantial reduction in genetic variation of up to 20%, and is likely both fast‐acting and pervasive [80, 86], though the relative importance of background selection and drift remains an open empirical question [87], and “narrow” gene‐culture coevolution is more typically interested in selective sweeps. In any case, the broader implication is that a substantial fraction of human genetic variation is dominated by nonselective processes.

Additionally, what many of these estimates have in common in that they rely on contemporary genomic data in making these complicated inferences. Conversely, Simon and Coop [72] decompose the contribution of gene flow, drift and selection to allele frequency shifts between both ancient (5000 years bp) and contemporary European genetic samples. They conclude that gene flow (especially from Yamnaya Steppe pastoralists) and drift account for virtually all changes, leaving only a marginal explanatory role for selection. Akbari et al. [88, p. 2] working with a similar data set to Simon and Coop, find “an order of magnitude more genome‐wide significant signals than previous studies,” but even they note that only about 2.35% of their observed allele frequency changes appear to be caused by directional selection—certainly a substantial number, but a far cry from being the dominant evolutionary force on the genome‐wide scale. In light of these results, a theory of gene‐culture coevolution that ignores neutral processes appears limited in its explanatory potential.

In fact, Simon and Coop [72] illustrate our point elegantly. Both the time frame and geography of their example broadly mirror the evolutionary context of the European lactase persistence example (i.e., Central Europe over the last 5000 years). Yet Simon and Coop find that this narrowly localized (at the genetic level) selective event, one of the strongest evidenced in the human genome, leaves virtually no detectable pattern on overall genetic variation. This is to say—narrow gene‐culture coevolution here explains the emergence of a particular phenotype, lactase persistence, but not the wider patterns of change in genetic variation. Those patterns appear instead to be largely shaped by an influx of Yamnaya steppe pastoralists into Europe, and the genetic legacy of those migrants [89, 90]. But what allowed this influx to happen? What cultural traits allowed the Yamnaya to spread across Europe in the first place (e.g., technological innovations such as wagons, or a pervasive culture of horse riding that enabled their nomadic pastoralism (see [91, 92])? And what interactions between sex, environment and culture may account for the remarkable divergence in sex‐specific genetic variation in Yamnaya lineages reported elsewhere [93, 94]? We do not claim that these questions go unanswered entirely, but they are typically not answered in the framework of gene‐culture coevolution. Broad gene‐culture coevolution does explore such questions, and by doing so may attempt to link patterns of cultural change and heterogeneity, which may themselves be characterized by cultural selection and adaptation, to the broad genetic shifts documented by [72]. Ultimately, ignoring how culturally evolved practices and institutions influence nonselective processes like drift and gene flow may overlook many interesting phenomena.

#### 2.2.1 Drift and Gene‐Culture Coevolution

Drift is a stochastic process of allele or trait frequency change that fundamentally depends on two factors: effective population sizes, and the strength of selection. In finite populations, smaller population sizes magnify the effects of sampling errors that arise from gametic reproduction or stochastic between individual variation in reproductive success. Additionally, since selection acts as a deterministic influence on allele frequency changes, its strength may limit the stochastic influence of drift (and vice versa). Both population size and strength of selection are likely to be profoundly shaped by culture [95]. Culture may intensify selection, both because cultural activities are capable of bringing about unusually rapid, consistent and heritable changes in environmental conditions relative to noncultural sources of selection [96], and because (at least in humans) cultural practices have led to striking increases in population size (discussed below). However, the opposite effect is also plausible, with culture leading to relaxed selection on genes. In domains where cultural and genetic traits covary and jointly influence phenotypes, changes in cultural variation may mask genetic effects, effectively shielding the underlying genetic variation from selection and increasing the role of drift in determining the dynamics of genetic evolution [6, 11, 14, 97, 98]. Interestingly, as the relevant mechanism here still primarily concerns changes in direction or magnitude of selection, this particular aspect of drift‐based gene‐culture coevolution can be construed as covered by “narrow” gene‐culture coevolution (e.g., [6]). In practice, however, investigating relaxed selection, while well‐documented within broader evolutionary anthropology (e.g., in the evolution of dental morphology [99, 100]), is not the usual aim of (empirical) gene‐culture coevolutionary study.

Furthermore, the relative strength of random genetic drift (over selection) is also a function of the effective population size of reproducing individuals [101, 102], which may also be subject to eco‐cultural influence. Subsistence transitions, for example, have likely facilitated substantial and rapid increases in population size and density since the mid‐to‐late Pleistocene by modifying environmental carrying capacity [103]. The genetic signatures of such subsistence transitions is evident even in some populations that transitioned from hunting and gathering to agriculture within the last millennium [104]. Notably, drift may affect both genetic and cultural variation (Figure 2), because stochastic variation in transmission is likely also to influence cultural evolution. Population size and other demographic factors, for example, likely play a role in the preservation of cultural variation and in the efficacy of cultural evolution to generate successful phenotypes [11, 105, 106, 107, 108, 109]. Additionally, in a reversal of the cultural masking mechanism described above, genetically evolved traits that functionally overlap previously learned ones may release some cultural variation from selective constraints [14, 110, 111]. In this way, a gene‐culture coevolutionary theory of drift still encompasses a fundamentally reciprocal model of cultural and genetic evolution.

#### 2.2.2 Example: Cultural Buffering Against Genetic Selection—Vitamin D‐Folate Theory of Skin Pigmentation Evolution, Material Culture, and Subsistence

Skin tone in humans is a well‐documented phenotype with remarkable geographic variation [112, 113, 114]. Biologically, it is a function of the distribution of melanosomes in the skin, intracellular organelles generated by pigment cells, which synthesize and store melanin pigments [115]. The most prominent adaptive hypothesis in regard to its evolution, the “Vitamin D” or “Vitamin D‐Folate” theory [116, 117, 118, 119], claims that this variation (and resulting geographical clines) is fundamentally driven by a trade‐off between two interacting selective processes, which jointly adjust levels of constitutive pigmentation to reflect (or, more precisely, absorb) environmental levels of UV radiation (UVR). Highly pigmented skin may be favored in UVR‐intense environments (i.e., equatorial latitudes) due to the photoprotective properties of eumelanin‐rich skin against the depletion of important light‐sensitive metabolites, including folate [120]. Conversely, more sparsely pigmented skin may be favored in UVR‐low environments (i.e., higher latitudes) as it allows allow for more efficient photosynthesis of cutaneous Vitamin D [117, 121, 122], generally thought to be a crucial nutrient in a wide range of physiological processes [123, 124], and which confers protection against certain diseases, such as rickets [125]. The resulting delicate balance between protective and permissive properties of human skin in relation to UVR gives rise to the high levels of phenotypic variation observed in contemporary human populations, and its apparent distribution along latitudinal clines [118].

More recently some researchers have suggested that the evolution of human skin pigmentation is best understood as a biocultural process, rather than a purely biological one [117, 126]. These biocultural approaches emphasize how cultural traits interact with both of the aforementioned phenotypes (UV protection and Vitamin D synthesis), modifying the respective strength of their selection and ultimately, the selection of the underlying genetic variation. For example, the cultural evolution of photoprotective material culture, such as various forms of clothing and their attendant production processes, may have modified whether pigmentation itself was necessary to protect against UVR [117, 127]. Also, Rifkin et al. [128] hypothesize that habitual skin application of ochre, a naturally photoprotective pigment made from clay, may have served early humans as a kind of early topical “sunscreen” (in addition to ritualistic use), allowing for dispersal into more UV intense habitats and reduced selective pressure from UVR.

An analogous biocultural argument can be made for Vitamin D synthesis. Cutaneous synthesis is not the only, or even primary, source of Vitamin D for the human body—much of it is dietary. Therefore, the relative fitness benefit gained from photoactive cutaneous Vitamin D synthesis may partially depend on the amount of Vitamin D in the diet. Culturally evolved subsistence practices modulate the dietary intake of Vitamin D and hence the relative importance of cutaneous production in maintaining “healthy” levels. This may explain why a shift to agricultural diets poor in Vitamin D at the start of the Neolithic may have exacerbated recent selection of depigmented skin [129, 130, 131]. In another example for the possible role of subsistence, strong facultative pigmentation (i.e., “tanning,” functionally convergent but mechanistically distinct from constitutive pigmentation) is highly prevalent in some contemporary aquatic hunter‐gatherer populations like the Inuit, despite their settlement in extreme latitudes [117]. This has been suggested as possible evidence for the role of dietary Vitamin D in the evolution of skin pigmentation, as many of these cultures have traditionally relied on an especially fish heavy diet [132]. Fish, particularly fatty fish, represents one of the primary dietary sources of Vitamin D [133], and its ready supply may have relaxed selection favoring depigmentation in some of these populations [117]. Further consideration of cultural factors such as dietary practices may shed more light on other examples where latitude alone is insufficient to explain phenotypic variation in skin coloration, including in Native American, European and Asian populations [134]. Further research in that regard could produce interesting examples of gene‐culture coevolution between cultural (dietary intake) and genetic (cutaneous production) influences on a crucial phenotype (Vitamin D synthesis), where cultural factors may act to buffer selection on genetic variation. We note that while such buffering processes are often understood on a phenotypic level, the power of gene‐culture coevolutionary inference here is to extend it to potential genetic signatures ‐ do we see increased genetic diversity at loci associated with skin pigmentation in populations with certain dietary traditions, or increased frequency of mildly deleterious alleles associated with the phenotype [135]? Using ancient genetic data, can we estimate the timing of a change in selective constraints, and determine whether it corresponds with changes in the cultural landscape? These are nontrivial, as‐of‐yet unanswered questions in the context of the evolution of skin pigmentation that an expanded gene‐culture coevolutionary approach could help formalize.

#### 2.2.3 Migration and Gene‐Culture Coevolution

Migration (or, in the genetic case, gene flow) is the other evolutionary mechanism that should be integrated into gene‐culture coevolutionary approaches. Perhaps one of the most remarkable features of the human species is its relatively rapid and pervasive dispersal out of Africa and around virtually the whole globe [136, 137]. The peopling of the world, now extensively traced through genomic as well as archaeological evidence, is a direct testament to human mobility and adaptability to novel environments, both of which are likely to be enabled and enhanced through cultural phenomena [138, 139]. While species obviously don't need culture to migrate, there is little doubt that cultural innovation, expressed in knowledge of migration pathways, navigation methods [140], modes of transportation (e.g., Anderson [141]), communication, subsistence and general problem‐solving capabilities have greatly enhanced, but also occasionally hindered, human migration, particularly in crossing major geographical barriers such as oceans, mountain ranges, and deserts [139].

This same set of cultural traits has made modern humans a deeply interconnected species. Indeed, contemporary genomics has repeatedly shown that extensive patterns of expansions followed by interpopulation gene flow and admixture have given rise to the remarkably continuous nature of human genetic variation [142]. This migratory tendency clearly affects both cultural variation (e.g., via demic or cultural diffusion) and its genetic counterpart (via gene flow and admixture). Demic and cultural diffusion are worth separating here, as cultural traits may “migrate” both in tandem with gene flow (via demic diffusion, i.e., the spread of holders of cultural traits [143]), or independently of it (via cultural diffusion, i.e., the spread of the cultural traits themselves through cultural transmission). Patterns of migration are likely deeply shaped by various eco‐cultural forces influencing the modes, pathways and impetuses of migration—like navigational traditions modifying migratory reach [138, 139], linguistic barriers or trade networks channeling the flow of migratory individuals [144], or postmarital residence norms regulating which sex disperses [145, 146]. Clearly, the role of culture in the dynamics of these processes is profound—yet its impact has largely been ignored in studies of gene‐culture coevolution.

#### 2.2.4 Example: Culture Shapes the Pathways of Migration—Trade Networks Shape Gene Flow in Melanesia, and Vice Versa

One compelling example of the gene‐culture coevolutionary dynamics of migration has been a well‐known part of the anthropological canon for the better part of the last century. In 1922, anthropologist Malinowski [147] published Argonauts of the Western Pacific, a now classic ethnography principally focused on the people of the Trobriand Islands in the Massim region off the eastern coast of Papua New Guinea (PNG). Much of this study painstakingly retraced the kula, a (roughly) ring‐shaped network of gift exchange spanning both linguistic and cultural boundaries across the entire region and adjoining the eastern tip of PNG with its outlying archipelagos. Kula societies exchange valuables, notably shell necklaces (soulava) and armbands (mwali), with specific trading partners on either side of their location in the ring, creating an intricate and seemingly historically deep system of circular gift exchange that remained in place, even flourished, long after European contact, and into the present day [147, 148, 149].

The functional and symbolic intricacies of the kula have received much attention over the decades following Malinowski's original description, and later anthropologists pointed out that it represents, in part, a kind of abstraction of deeper intercultural networks of trade and alliances existing in the region [148]. All manner of resources are said to have followed the flow of the kula ring shells, trade goods and people alike. Similarly, the concept of the kula itself, and the associated rites and myths, seem to have spread and been reinforced across the region in a clear example of cultural transmission [150]. Additionally, the configuration and constancy of the kula ring were likely shaped by a mosaic of cultural and ecological factors, including winds, island ecology and maritime technologies—pointing toward the deep importance of a whole range of culturally evolved factors [150].

What might a gene‐culture coevolutionary hypothesis for the kula look like, if gene flow were the primary focus? We might expect populations connected through the network to be more closely related genetically than geographic distance alone would suggest, as the kula may facilitate gene flow (in the form of migration and intermarriage) and subsequent homogenization between these populations. While population genetic studies of the region are few and far between, the two studies that do exist render such a connection highly plausible. A study by van Oven et al. [151] examined patterns of paternally inherited Y‐chromosomes (NRY) and maternally inherited mitochondrial DNA (mtDNA) across the Massim region. This study included both island populations that traditionally participate in the kula, as well as some that do not (although such categories are difficult to establish given the intrinsically flexible nature of the kula emphasized by anthropologists). Two results stand out in evaluating the potential role of the kula in directing gene‐flow in the region: first, both NRY and mtDNA data suggested that local genetic structure is best explained by a rough division of the region into a northwestern group (consisting of islands that traditionally participate in the kula), a southeastern group (consisting of islands that traditionally do not participate), and Rossel Island (a traditionally endogamous culture on the very southeastern tip of the Massim). This finding implicates the network as a mechanism of differentiation.

The second finding, perhaps even more remarkable, comes from examining NRY and mtDNA data separately. Here, evidence suggests that kula‐trading populations show relatively clear population differentiation (roughly as predicted by the isolating effects of distance) in their mtDNA, but not their Y‐chromosomal DNA. This is to say that male‐inherited genetic diversity is much more genetically homogeneous across the kula‐ring than is female‐inherited genetic diversity. It is important to consider here that kula voyages were traditionally mainly made by men [148], so a plausible hypothesis may be that kula‐mediated male gene‐flow has given rise to these patterns. Strikingly, the few Massim region cultures included in cross‐cultural data sets like the Ethnographic Atlas are coded as virilocal (i.e., effectively patrilocal, suggesting female migration), in line with the overwhelmingly patrilocal traditions of wider PNG [152] ‐ but the pattern observed in the Massim runs exactly counter to the expectation for patrilocal groups [145, 153]. This renders postmarital residence norms an unlikely alternative explanation. Overall, van Oven et al. [151] make a strong case for role of the kula in affecting the genetic structure of the Massim.

Subsequently, Liu et al. [154] analyzed genome‐wide data of 192 individuals across 15 groups to further elucidate the genetic structure of the Massim region. Instead of genetic distance, they examine patterns of IBD sharing (shared tracts of DNA between individuals that indicate common descent, often used to infer migration) to interrogate a potential role for the kula in shaping the regions genetic structure. They find higher IBD sharing among kula‐participating islands than among those excluded from the traditional network, indicating, again, a potentially facilitative role of the exchange network. Notably, however, they infer for these patterns to have time depth of thousands of years, predating archaeological evidence of the kula by some time [150]. They offer two nonexclusive explanations for the observed pattern: first, it is likely that although the kula tradition itself may be a relatively recent phenomenon, it reflects a network of trade and alliance partners of considerable deeper chronological depth [155, 156]. Second, and particularly appealing to the reciprocal nature of coevolutionary approaches, Liu et al. [154] speculate that it may, in part, have been the connectivity, shared ancestry and genealogical connection between the islands of the Massim region that gave rise to the kula system—a case, perhaps, of genetic affinity leading to a self‐reinforcing cultural practice (or genes influencing culture in an unusual and indirect way), as the resulting gift exchange network in turn may have facilitated gene flow that could reinforce those same kinship ties. This would serve as a striking illustration of nonadaptive gene‐culture coevolutionary dynamics. These findings could also inform future work on the cultural side of gene‐culture coevolution: recent advances in cultural evolutionary theory call for increased focus on viewing cultural populations as information networks [157, 158]. Genetic work like that done in the Massim can elucidate, empirically, how such networks arise and change over time, perhaps even in ways that are invisible to archaeology (as evidenced here by the significant deeper time depth of genetic linkages over Kula evidence), and thus set the stage for better empirical models of the diffusion and differentiation of culture based on migratory links.

#### 2.2.5 Broader Than “Broad”: Further Candidate Mechanisms for Gene‐Culture Coevolution

We are of course not proposing a final, all‐encompassing theory of gene‐culture coevolution—quite the opposite. Although this expanded approach privileges selection, drift and gene flow as the primary evolutionary mechanisms of gene‐culture interaction, it need not do so. Indeed, there are any number of other mechanisms that could be, and have been, construed to be evolutionary processes—most notably mutation, but also recombination or assortative mating (e.g., Mayr [159] Posada et al. [160]). It is conceivable, even likely, that cultural influences may play a role in those domains as well, though we will cover them only briefly here, as the work on these domains in human genomics is nascent and has not yielded clear examples like the role of culture in shaping drift and migration.

To give an example, however, considering the well‐established role of parental age on the human germline mutation rate [161, 162], it is possible that kinship norms regulating age‐at‐marriage or age‐at‐first‐birth play a role in the emergence of population‐level differences (either across space or time) in mutation rates (but see Gao et al. [163]). Other ostensibly cultural effects on the human mutation germline mutation rate are more sinister, as work on the effects of nuclear weapons testing [164] and the Chernobyl disaster [165] demonstrate (note that we are are not necessarily suggesting those are topics ripe for gene‐culture coevolutionary analysis). A similar logic may even be applicable to the study of population variation in recombination rates. Though variation is subtle between human populations [166, 167], the forces that give rise to such differences are not yet fully understood, and may, for instance, involve differential selection on genes that shape recombination, like PRDM9 [168], which in turn may be driven by culturally‐modified environmental conditions such as pathogen challenges [169]. But beyond heritable genetic variation (in fact, heritability of recombination rate variation is “only” around 0.3 [170]), empirical evidence points toward remarkable plasticity in recombination rates in response to certain environmental conditions, including temperature [171] and nutritional stress [172]. Strikingly, like mutation rates, recombination rates also seem to be related to maternal age in humans [173].

**Figure 3:**
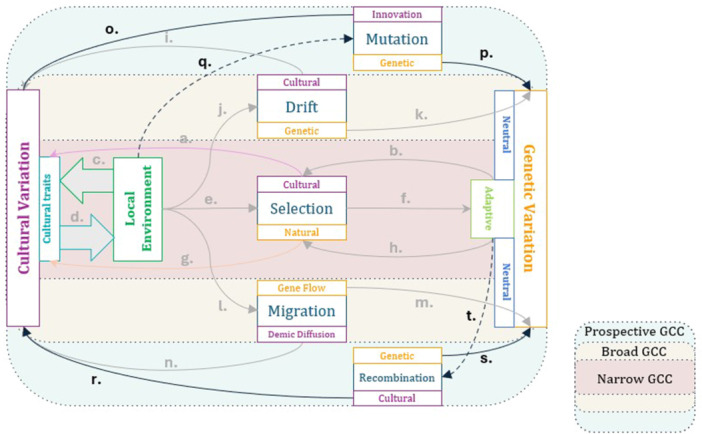
Potential areas of future research on gene‐culture interactions. There might be gene‐culture coevolutionary processes beyond those outlined in previous figures, concerned with candidate mechanisms that we have not discussed. Some plausible causal links, suggested from adjacent literature, are represented through dashed lines. (o) Mutational processes in cultural evolution, that is, innovation, generating novel cultural variation, much like, (p) mutational processes in genetic evolution generating novel genetic variants, (q) the eco‐cultural environment shaping both of those processes, for example, through social network structures favoring innovation, or through reproductive processes affecting the average germline mutation rate across populations, (r) cultural recombination generating new cultural phenotypes by recombining existing cultural traits across lineages (e.g., Creanza et al. [174]), (s) genetic recombination generating novel haplotypes, (t) evolved genetic traits affecting the speed and extent of recombination for both genetic variation (e.g., recombination rate evolution) and cultural variation (e.g., evolved cognition).

All of these factors—selection, environmental stress, maternal age—may have cultural components that can help us understand variation in the mutation and recombination landscape and, perhaps, their broader genomic footprint. While we will not expand further on this here, in the future, the argument could be made that work on those kinds of questions may fit within a gene‐culture coevolutionary framework (Figure 3). In a way, gene‐culture coevolution models may be useful here *because* the work is nascent, and could embrace a coevolutionary perspective from the start.

### 2.3 Gene‐Culture Coevolution Reconsidered

Having laid out a case for this expanded definition of gene‐culture coevolution, it is worth discussing how we believe this approach differs from some other attempts at organizing related research, starting with more recent work. Waring and Wood [98] specify three “modes” of gene‐culture coevolution trait‐pair coevolution (more in line with narrow‐sense coevolution), trait‐system coevolution, and system‐system coevolution. “System” here refers to inheritance systems, that is, cultural or genetic inheritance. Trait‐pair coevolution describes the “classic” reciprocal changes between a cultural trait and a genetic one, that is, a cultural trait altering the fitness of a certain genetic trait, and vice versa. Trait‐system coevolution describes the influence that single traits in one domain can have on the entire inheritance system in the other. Finally, system‐system coevolution describes a scenario where having two separate (and mechanistically distinct) pathways for (adaptive) evolution may lead to one system “crowding out” the other. While very useful and encompassing many gene‐culture interactions that might be included in the gene–culture coevolutionary framework, this approach is more concerned with categorizing the respective targets (or levels) of reciprocal change, rather than the evolutionary mechanisms through which such targets interact. Therefore, it represents an approach that is orthogonal to our focus on mechanisms.

A classic attempt at categorizing gene‐culture interactions comes from Durham [6]. In fact, Durham [6] utilized a “narrow”/”broad” gene‐culture coevolution terminology similar to that introduced here. However, Durham's approach does not include the role of what he calls “non‐conveyance forces” in both genetic and cultural evolution, which include mutation, innovation, migration, and drift. Consequently, Durham's scheme is exclusively focused on reciprocal fitness changes between genetic and cultural traits, and the directionality of these changes (i.e., degree of concordance between the cultural and genetic fitness effects of relevant traits)—leaving out “non‐conveyance forces” entirely.

Following their earlier research on whales suggesting that culture could shape neutral genetic variation and account for low genetic diversity [175], Whitehead et al. [30] proposed a broader scheme of gene‐culture coevolution that is roughly in line with our own. Like those authors, we propose that defining gene‐culture coevolution in this broad sense, which includes those “non‐conveyance forces” and the role that cultural and genetic variation play in shaping them, gets to the core of what we suspect most gene‐culture coevolutionists are interested in—namely, understanding the causal roles that culture and cultural evolution play in shaping genetic evolution in general, not just adaptive genetic evolution. One might argue that our re‐framing of coevolution diminishes the utility of drawing parallels to evolutionary ecology, but similar argument to ours have recently been made in that context as well [26, 176], showing that endeavors to rethink coevolution are not limited to our human‐focused gene‐culture perspective.

Despite our focus on mechanistic and conceptual inclusivity, however, it is important to consider possible boundaries for this broader definition of gene‐culture coevolution. Incidentally, drawing such boundaries was the exact impetus for the “traditional” definitions of coevolution (e.g., [21]), which we introduced in the opening paragraphs and have moved to revise in the present article. We agree, as Thompson [23, p. 181] notes in his breakdown of the use of the term “coevolution” in evolutionary biology, that the word potentially “loses its utility when it is applied so broadly that all possibility of analysis of the mechanisms of reciprocal change is lost.” And while we do extend the term beyond how it is typically used, this concern appears equally valid in the case of gene‐culture coevolution. A definition must be bounded, or it runs the risk of being diluted to the point of incoherence. For the purposes of clarity, it is therefore useful to reconsider some of those previous misgivings with the imprecise use of “coevolution” in evolutionary biology [21].

For example, we believe it is important to distinguish between evidence of interaction and mere mutualistic congruence, that is, patterns of correspondence that are not due to reciprocal influence. Specifically, one process that may lead to correspondence between genetic and cultural variation derives from the idiosyncrasies of a dual‐inheritance system, and may help demarcate what does not constitute gene‐culture coevolution. Consider a cultural trait that is transmitted vertically across generations, from parent to child. Through time and space, the history and spread of this trait may almost directly mirror the history and spread of genes, as it travels in parallel with genetic information [4]. Affected equally by demographic change, migration, and founder effects, geographical patterns of genetic and cultural variation may come to correspond, yet nowhere in this hypothetical scenario do the traits actually interact, in the sense of influencing each other's transmission—they are merely inherited together. As noted early on by Cavalli‐Sforza and Feldman [11], this renders such correlations between cultural and genetic variation spurious rather than causally meaningful. In the study of genetic and cultural variation, the possibility of such “parallel transmission” scenarios is most clearly exemplified by the relationship between linguistic and genetic lineages. Numerous studies, for example constructing co‐phylogenies from linguistic and genetic data, have shown that there are clear patterns of similarity between linguistic and genetic diversity in humans [177, 178, 179, 180]. These patterns might stem from parallel transmission of language and genes—via processes like “local codiffusion” (i.e., concurrent horizontal transmission) or simply be due to the aforementioned parallel vertical transmission [177]. Such phenomena constitute a useful boundary for gene‐culture coevolution—if culture and genes merely travel together, but do not influence each other's spread or dynamics, this does not constitute gene‐culture coevolution. Note that in practice, such a pattern may however be difficult to distinguish from patterns generated by reciprocal adaptation when observed in contemporary data alone (an issue known as equifinality [181]).

Causal interaction through any evolutionary mechanisms, even if it is (seemingly) unilateral, should be the defining property of gene‐culture coevolution. It is worth noting here that the gene‐language coevolution literature indicates that this hypothetical “perfect parallel transmission” scenario is likely to be rather transient in human populations, possibly because language often evolves much faster than genes. Over longer time spans, as Barbieri et al. [177] point out, linguistic and genetic lineages may give different estimates of divergence times, leading the authors to assert that preceding genetic diversification may trigger later cultural diversification, whereas early linguistic diversification may cause barriers to gene flow which affect genetic structure. Both of these scenarios, in turn, would be coevolutionary mechanisms that satisfy our simple criterion of nonindependent co‐inheritance. Another interesting case is that of gene‐culture hitchhiking, where parallel transmission of genetic and cultural variation leads to incidental “pseudolinkage” between the two, so that subsequent selection in one domain leads to reduction in variation in the other [93, 182], which in turn may lead to interesting and understudied coevolutionary dynamics and effects like selective interference. Nevertheless, perfect parallel transmission without linkage should serve as a useful null model for empirical investigations of gene‐culture coevolution where correspondences between genetic and cultural variation are observed. Such patterns are still interesting and meaningful—for example, in the interpretation of genome‐wide association studies (GWAS), which may be confounded by the independently co‐inherited genetic and cultural traits whose relationship is not actually causally relevant [183]. In empirical data, however, such a null‐model may be difficult to discern, and we will discuss such challenges to the gene‐culture coevolutionary approach further below.

### 2.4 The Utility of a Broadened Approach to Gene‐Culture Coevolution

By focusing on mechanisms of evolutionary change, we hope the framework proposed here will be useful in two ways: looking back and looking forward. First, we believe this expanded definition may serve to reframe a significant amount of insightful existing research as being perfectly consistent with, even supportive of, gene‐culture coevolution as a theoretical framework. This study, despite not being explicitly framed as gene‐culture coevolution, perhaps partly due to its focus on nonselective phenomena, has done much to showcase the deep influence of cultural phenomena on human genetic variation. Its full extent deserves a review in itself, but a nonexhaustive list may include genetic structure being shaped by linguistic boundaries [144, 184, 185], postmarital residence norms [145, 146, 153], subsistence transitions [104], assortative mating [186, 187], culturally determined social stratification [188, 189], or endogamy and consanguineous marriage norms [190]. We stress that this study stands by itself, without strictly necessitating incorporation into some grander framework of gene‐culture interactions. But its common exclusion from the discourse around gene‐culture coevolution unnecessarily diminishes the contribution this study has made to our understanding of how culture, and cultural evolution, has played an “ultimate” role in shaping genetic changes in our species. In a recent retrospect on the study of cultural evolution [191, p. 4], point out that “very few cases of gene‐culture coevolution have been worked out in detail.” This is true even with the inclusion of nonadaptive cases of gene‐culture interactions. But the evidence base does grow substantially when we acknowledge these examples, as we believe we should.

Second, more interesting perhaps, then, is how a gene‐culture coevolutionary framing may benefit this type of work, and work in evolutionary anthropology more broadly, going forward. Looking to the future, we hope that our framework may open new avenues of theoretically motivated empirical research on gene‐culture coevolution. By using a broader range of well‐established evolutionary mechanisms or forces as the organizing principle for understanding gene‐culture interactions, broad gene‐culture coevolution may provide a promising way of detecting novel reciprocal influences of cultural and genetic variation. As we discussed in our introduction, gene‐culture coevolution shares with wider cultural evolutionary theory a solid grounding in quantitative theory [3, 11]. Indeed, gene‐culture coevolution arguably draws on an even wider set of underlying theory, as it spans both “cultural” and “biological” modes of evolution. However, we also believe the two disciplines also share a certain vagueness in which theoretical and empirical work are connected in practice [192]. Empirical gene‐culture coevolutionary work, fundamentally, seeks to infer causal effects of cultural traits on genetic structure, and vice versa. As such, it fits neatly with the causal inference frameworks advocated for in cultural evolution [192, 193].

We defer to these papers as a better, deeper introduction into the methodological frameworks of causal inference. To give only a brief overview, in their introduction to causal frameworks for cross‐cultural research (a context that closely matches that of much empirical gene‐culture coevolutionary work), Deffner et al. [193] introduce four prerequisites for causal frameworks: (a) a target variable or value of interest, that is, the estimand, (b) a generative model of the causative influences on that estimand, (c) a generative model of how populations may differ, especially in regard to influences on the estimand, and (d) an analysis strategy for subsequent statistical inference. We believe a broad approach to gene‐culture coevolution can inform such causal models on all four points. Looking beyond selection, population genetics provides ready‐made novel estimands for “broad” gene‐culture coevolutionary inference, for example IBD‐sharing patterns that can quantify migration potentially influenced by cultural practices [154]. More importantly, a broader approach enables richer generative models, commonly visualized as direct acyclical graphs (DAGS [194]), which encode potential causal relationships. Indeed, the visualizations included in the present paper essentially map some of these possible causal links. This approach may help researchers specify the “rules of engagement” between genes and culture, formalizing both cultural mechanisms (e.g., how matrilocality affects migration) and genetic signatures (e.g., sex‐biased gene flow patterns), while also helping to identify potential confounders and deeper, reciprocal causal interactions. Integrating cultural evolution, behavioral ecology and population genetics, we can develop better theoretical models and predictions that inform downstream empirical research design, or at the very least assist with the generation of novel hypotheses. If the aspiration of gene‐culture coevolution as a field is to understand culture's “ultimate” role in genetic evolution [191], we should utilize the full inferential toolbox of adaptive and neutral evolutionary theory, moving beyond estimates from observed associations toward a deeper causal understanding.

We of course harbor no illusions that even good causal models and well‐founded hypotheses would make empirical gene‐culture coevolutionary research suddenly straightforward. Working necessarily across the domains of genetic and cultural data, both must be treated with the care required by their respective complexities. Indeed, an empirical science of gene‐culture coevolution will require a synthesis of anthropology, archaeology, cognitive sciences, ecology, developmental and evolutionary biology, genetics, genomics, history, and mathematics—with each discipline contributing uniquely to the resulting work (see also Zeder [195]). Burgeoning methodological advancements integrating cross‐cultural and archaeological data with genetic research show promise (e.g., [50]), but significant challenges remain. Perhaps most daunting is that gene‐culture coevolution's most remarkable potential manifestations, for example, the emergence of cooperation or the peopling of the world, demand dynamic rather than static causal models, potentially spanning millennia in time and the entire human species in scope. This raises challenging fundamental questions: How do we model these dynamic causal processes? Are they uni‐ or bidirectional? What data enable robust statistical inference, and is such data even available? All these things ought to be carefully considered in the potential creation of new gene‐culture coevolutionary approaches. Data availability, in particular, may be a tangible bottleneck for many researchers contemporary cultural and genetic data from diverse populations is often plentiful, but inference based on contemporary data alone may suffer from equifinality [181]. For example, distinguishing between cultural variation “shielding” against selection, and the simple absence of such selection, poses a considerable challenge if based on contemporary genetic data alone. Advances in archaeology and ancient DNA research promise to address these limitations [196, 197], but these data sources present their own methodological challenges [198, 199, 200]. While we remain optimistic about the utility of our broadened gene‐culture coevolutionary framework, we caution against assuming we are just “some more data points” away from definitively illustrating culture's purportedly transformative role in human evolution.

## 3 Conclusions: A Science in Progress

Overall, we believe our approach dispels the notion that gene‐culture coevolution is strictly a rival theory to other approaches in the evolutionary human sciences. Rather, we view it as an overarching, guiding framework, the constituent parts of which can be investigated using any number of approaches. This includes human behavioral ecology (e.g., examining the relationship between cultural traditions and ecology [32]), psychology (examining how cross‐cultural cognition and the cognitive machinery of social learning emerge in the first place [201]), and cultural evolution itself. Other recent work has similarly argued for some conceptual rearrangement and synthesis [202].

There are a number of coevolutionary phenomena that fall within our broad conceptual framework, but (largely) outside of the scope of what has been presented here. In evolutionary biology, coevolution is often an inter‐specific process [21]—and human culture certainly has dramatically influenced the evolution of many species with which we share environments and ecosystem, including both animal and plant domesticates [203]. This influence of human culture on nonhuman genetic evolution, and vice versa, is causally intricate and likely ubiquitous (e.g., via artificial selection [204, 205]). In a striking example that recapitulates the textbook case of gene‐culture coevolution, for instance, there is some evidence to suggest that European dogs evolved a lactase persistence‐like phenotype in roughly the same time frame as humans [206]. There is also evidence to suggest that the European subsistence transition to agriculture was associated with adaptation to more starchy diets in the domesticated dogs of the region [207, 208]. Such interactions doubtlessly open many avenues for scientific inquiry, including how the spread of certain cultural traits may correspond to the genetic structure of many domesticated organisms. The same goes for considerations of gene‐culture coevolution beyond the human domain. The study of “animal cultures,” socially acquired behavioral traditions in nonhuman animals, is a growing science [209, 210, 211]. There is now rapidly accumulating evidence that interactions similar to those that that mark human cultural and genetic evolution may lead to gene‐culture coevolutionary phenomena in nonhuman animals [30], spanning the animal kingdom from cetaceans to insects [175, 212]. The applicability of a gene‐culture coevolution framework outside of human evolution should help to integrate the theory into the broader canon of evolutionary biology.

We hope that the current discussion, as well as the examples we elected to illustrate it, goes some way to make the case for a broader science of gene‐culture coevolution that would encourage a more inclusive analysis of the potential causal interactions between genes and culture. Empirical investigation of gene‐culture coevolution remains a fledgling scientific effort, but our hope is that expanding the rigorous theoretical framework in which gene‐culture coevolution was originally conceived may prove to be useful for the study of human biocultural evolution across disciplines, including anthropology—whose nuanced understanding of the breadth and depth of human culture is central to such efforts (see also Wiley and Cullin [213]). Ultimately, it will surely take a concerted, cross‐disciplinary effort to answer this one simple question: how, and to what extent, have humans shaped their own evolution?

## Data Availability

Data sharing not applicable to this article as no data sets were generated or analysed during the current study.
